# Economic evaluation of a group-based exercise program for falls prevention among the older community-dwelling population

**DOI:** 10.1186/s12877-015-0028-x

**Published:** 2015-03-26

**Authors:** Kendra McLean, Lesley Day, Andrew Dalton

**Affiliations:** Melbourne School of Population and Global Health, The University of Melbourne, Victoria, Australia; Monash Injury Research Institute, Monash University, Victoria, Australia; Strategic Research Centre, Population Health, Deakin University, Victoria, Australia; Bentleigh Bayside Community Health, PO Box 30, Bentleigh East, 3165 Victoria Australia

**Keywords:** Accidental falls, Cost effectiveness, Older adults, Exercise

## Abstract

**Background:**

Falls among older people are of growing concern globally. Implementing cost-effective strategies for their prevention is of utmost importance given the ageing population and associated potential for increased costs of fall-related injury over the next decades. The purpose of this study was to undertake a cost-utility analysis and secondary cost-effectiveness analysis from a healthcare system perspective, of a group-based exercise program compared to routine care for falls prevention in an older community-dwelling population.

**Methods:**

A decision analysis using a decision tree model was based on the results of a previously published randomised controlled trial with a community-dwelling population aged over 70. Measures of falls, fall-related injuries and resource use were directly obtained from trial data and supplemented by literature-based utility measures. A sub-group analysis was performed of women only. Cost estimates are reported in 2010 British Pound Sterling (GBP).

**Results:**

The ICER of GBP£51,483 per QALY for the base case analysis was well above the accepted cost-effectiveness threshold of GBP£20,000 to £30,000 per QALY, but in a sensitivity analysis with minimised program implementation the incremental cost reached GBP£25,678 per QALY. The ICER value at 95% confidence in the base case analysis was GBP£99,664 per QALY and GBP£50,549 per QALY in the lower cost analysis. Males had a 44% lower injury rate if they fell, compared to females resulting in a more favourable ICER for the women only analysis. For women only the ICER was GBP£22,986 per QALY in the base case and was below the cost-effectiveness threshold for all other variations of program implementation. The ICER value at 95% confidence was GBP£48,212 in the women only base case analysis and GBP£23,645 in the lower cost analysis. The base case incremental cost per fall averted was GBP£652 (GBP£616 for women only). A threshold analysis indicates that this exercise program cannot realistically break even.

**Conclusions:**

The results suggest that this exercise program is cost-effective for women only. There is no evidence to support its cost-effectiveness in a group of mixed gender unless the costs of program implementation are minimal. Conservative assumptions may have underestimated the true cost-effectiveness of the program.

**Electronic supplementary material:**

The online version of this article (doi:10.1186/s12877-015-0028-x) contains supplementary material, which is available to authorized users.

## Background

Falls pose a major public health concern globally [1]. Approximately 1 in 3 people aged over 65 living in the community fall each year, a rate which increases with age [2]. Falls, fall-related injuries and subsequent fear of falling can have a significant impact on health-related quality of life and function, resulting in loss of independence among older people [3]. The medical costs of fall-related injuries increase rapidly with age and are 2 to 3 times higher for women due to their higher risk of osteoporotic fractures, particularly hip fractures [4]. Developing and implementing cost-effective programs to prevent falls among older people is of utmost importance given the ageing population and associated potential for increased costs of fall-related injury over the next decades.

Exercise is currently the only proven falls intervention appropriate for population level delivery [5] and is the falls intervention subject to most economic evaluations [6]. Many previously reported economic evaluations of exercise programs to prevent falls are founded on the home-based “Otago Exercise Program” [7-11]. Whilst this program demonstrated a reduction in falls rate of between 30% and 46% compared to routine care [9-11] it failed to achieve cost-effectiveness or monetary savings apart from in a sub-analysis of people aged over 80 years [9]. Economic evaluations [7,12] based on a systematic review by the Cochrane Collaboration [13] have explored the impact of falls on health-related quality of life. One evaluation included group-based exercise and demonstrated cost-effectiveness for high risk populations only [12].

There are limited published economic evaluations of group-based exercise for falls prevention, a more suitable format for population level delivery. The purpose of this study was to determine cost-effectiveness of one group-based exercise program that has been proven effective in reducing falls [14,15].

## Methods

### Study overview

The objective of this evaluation was to determine whether a group-based exercise program is cost-effective compared to routine care to prevent falls among the older community-dwelling population. A decision analysis was performed using a decision tree model. The evaluation was conducted from a healthcare system perspective as significant drivers of cost are within the healthcare sector. As health-related quality of life is an important outcome of falls prevention, a cost-utility analysis (CUA) was undertaken as the primary analysis. This analysis also allows broader comparisons to other healthcare programs. Secondary cost-effectiveness analyses (CEA) include the incremental cost per fall, injurious fall, and fracture averted. Measures of falls, fall-related injuries and resource use for the economic evaluation were obtained directly from trial data over an 18 month time horizon corresponding to the duration of follow-up. This was supplemented by literature-based utility measures.

All costs were identified from the perspective of the healthcare system and converted from 2010 Australian Dollars to British Pound Sterling (GBP) using 2010 purchasing power parities. All costs and consequences were discounted at a rate of 3% in the base case analysis, as recommended by the Panel on Cost-effectiveness in Health and Medicine [16].

### Intervention

This evaluation was based on the results of a previously published randomised controlled trial of the “NoFalls” exercise program [14,15]. The exercise program consisted of a weekly one hour group-based exercise class for 15 weeks, supplemented by daily home exercises. The class consisted of graded exercises to improve flexibility, leg strength and balance [14,17]. The comparator is routine care and activity, considered standard care.

Approval for the “NoFalls” trial was obtained from the Monash University's Standing Committee on Ethics in Research Involving Humans. The “NoFalls” trial took place in metropolitan Melbourne, Australia with a community-dwelling population aged over 70 years, recruited from the electoral roll. One thousand one hundred and seven participants were randomised, with 17 withdrawing immediately post-randomisation. Five hundred and forty-one participants were randomly assigned to receive the exercise intervention and 549 to receive no exercise intervention. The original “NoFalls” trial also investigated the effect of home hazard management and vision improvement on falls prevention in a full factorial design. However these interventions on their own failed to show a significant effect so have been excluded from this evaluation. During the 18 month follow-up period 9.5% of participants withdrew and 1.4% deceased, however data was provided for at least 1 month by 98.5% of randomised participants. The study participants at baseline had a mean age of 76.1 years and were 59.8% women. Baseline demographics between groups were similar [14]. Participants in the exercise group fell at a rate 21% lower than those in the routine activity group (IRR:0.79, 95% CI 0.67 to 0.94) [15].

### Analytical framework

**Figure 1:**
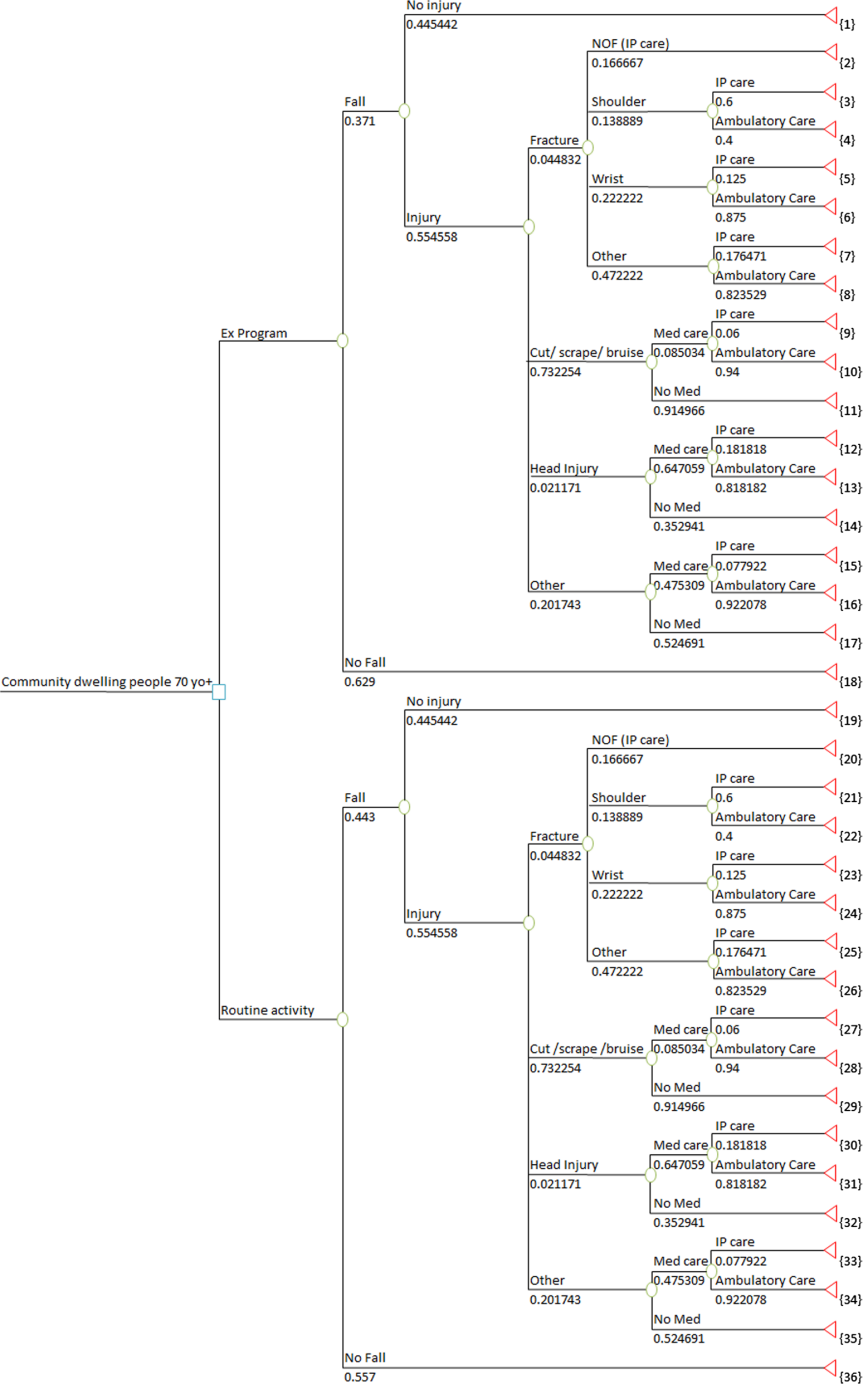
**Decision tree.**

A decision tree model was used in the decision analysis to establish potential pathways of participants (Figure 1). A negative binomial regression model was used to calculate the rate of falls in each group and to determine the rate of injury if a fall occurred, using PASW Statistics 18 [18]. Negative binomial regression is recommended for analysis of falls count data as falls are recurrent and data over-dispersed (i.e. variance is greater than the mean) [19,20]. The model involved entering the surveillance period as the offset variable, number of falls as the dependent variable, and the intervention as the categorical variable. Additionally the number sustaining an injury if a fall occurred was substituted as the dependent variable. Interactions with age and gender were also explored. The rates obtained from the analysis were converted to probabilities over 18 months for use in the economic evaluation.

All analyses were performed by intention to treat with statistical significance set at p ≤ 0.05. The decision analysis was performed using Microsoft Excel 2007 with Risk Solver Platform V10.0 [21]. A sub-group analysis of women only was performed in keeping with suggestions that women have a higher risk of falls and related injury [1,4]. Results are reported in the form of an incremental cost-effectiveness ratio (ICER), calculated by dividing differences in costs by differences in effects of intervention and comparator.

#### Secondary CEA

Falls were recorded prospectively for up to 18 months via monthly post-card calendar to optimise accuracy and injuries self-reported via telephone interview. Good agreement has been demonstrated between self-report and medical records, and if anything self-reporting tends to result in under-reporting [22]. A fall was defined as “losing your balance such that your hands, arms, knees, bottom or body touch or hit the ground or floor” [14]. Injuries were classified as a fracture, cut/scrape/bruise, head injury or other injury. Falls data was obtained for 92% of possible surveillance points over 18 months.

### Health-related quality of life (HRQoL)

Health-related quality of life is measured on a scale of 0 (death) to 1 (perfect health) using a utility instrument which measures individual strength of preference between alternative health states [16]. The EuroQol (EQ-5D) is a valid and reliable instrument widely used to obtain utility weights for fall-related conditions in older populations [23,24]. Utility weights are combined with time spent in different health states to calculate quality-adjusted life years (QALYs) [16]. An utility instrument such as the EQ-5D was not used during the “NoFalls” trial. Therefore utility weights from this instrument have been sourced from published results for similar populations (Table 1).

**Table 1:** **Utility value of health states**

**Utility**	**Value**	**Utility applied to 18 month model**	**Source**
No fall	1	1	
Fear of falling	0.94		Iglesias [25] (EQ-5D)
Fall	0.997		
Fall – including proportion fear of falling		0.99336 (overall)	Probability of FOF: Freidman [26]
		0.98466 (women)	Murphy [27]
Hip fracture		0.73	Peasgood [28]
Year 1	0.7		(95% CI: 0.64 - 0.77) (EQ-5D)
Year 2	0.8		(95% CI: 0.68 - 0.96) (EQ-5D)
Shoulder fracture	0.94	0.94	National Osteoporosis Foundation [29] (EQ-5D)
Wrist fracture	0.956	0.969 (overall)*	Peasgood [28]
		0.966 (women)	(95% CI: 0.86 – 1.00) (EQ-5D)
Other fracture	0.94	0.958 (overall)	National Osteoporosis Foundation [29] (EQ-5D)
		0.955 (women)	

*Disutility of a wrist and other fracture is applied for 12 months and for the remaining 6 months of the 18 month time horizon a proportionate disutility of fear of falling is applied.

Literature-based utility estimates for injuries other than fractures are limited, therefore equivalent utility was applied to a fall unless a fracture sustained. Utility was applied evenly to a fracture regardless of management. Although inpatient care may result in higher utility loss this could not be differentiated from the literature. Whilst research continues into the social value of a QALY and how it varies with age [30], a baseline utility of 1 was allocated to a non-faller indicating normal age-specific health. In reality the baseline utility of the elderly is below this value and it has been suggested that using a baseline utility value of 1 provides an overestimate and age/sex norms that exist for instruments such as the EQ-5D should be used instead [31]. This then raises a concern that the value of health benefit such as the QALY depends on when it arises and who receives it [32], making people with higher baseline utility apparently more deserving of a QALY than others. Applying a value of 1 as the baseline utility is more equitable when it comes to making comparisons of programs and their incremental QALY gain.

The QALY calculation assumes that falls reduction applies to the 18 month follow-up after which participants return to their pre-treatment falls risk. There is no evidence supporting longer term effects once an exercise program has ceased [33]. Evidence also suggests that following a fracture there is a return to the previous level of utility within 1 to 2 years [28].

The probability of developing fear of falling (FOF) was obtained from studies with similar populations [26,27]. Persistence of FOF is recognised [25] therefore disutility associated with FOF was applied for the 18 month period for those who fell. FOF was applied equally to both groups, although there is evidence to suggest that exercise interventions can reduce FOF in this population [34]. A higher rate of FOF has been associated with more injurious falls [35], but as no clear incidence is available the probability of FOF was applied evenly. Probability of developing FOF was increased in the women only sub-analysis in accordance with the evidence available [26,27]. The probability of developing FOF and utilities applied are conservative, most likely underestimating the true impact of the exercise program.

### Costs

#### Program implementation

Program costs included labour, equipment, venue hire, music and consumables (Additional file 1: Table S1). Research protocol driven costs were excluded. The cost of training staff was excluded as their classifications indicate they are already qualified for their role.

An Allied Health Assistant (AHA) was designated as group instructor in the base case analysis due to their prevalence in Australian healthcare settings. A fitness instructor could run this program at a cheaper hourly rate and was included in the sensitivity analysis. Labour was valued by hourly wage plus 50% on-costs. For each 1 hour session 1.5 hours labour was allocated, and 5 hours labour per group for screening potential participants.

The costs of recruiting each group for this program are unknown. An estimate of advertising was included in the base case analysis. Advertising in a local newspaper is unlikely to be required but is included in a sensitivity analysis.

#### Cost of falls

Resource utilisation was self-reported via telephone interview following a fall for 93% of reported falls, but could not be verified against healthcare records as permission was not obtained for this trial. General Practitioner consultations, ambulance services, Emergency Department visits and hospital admissions were included as fall-related costs (Additional file 2: Table S2). Outpatient utilisation of Allied Health, investigations, medication and specialists was not available from the trial. As hospital inpatient services are major cost drivers following falls in this age group [9,36,37] exclusion of outpatient services was not expected to alter cost-effectiveness substantially, but was explored in a sensitivity analysis.

Consultation with a General Practitioner was recorded but not occasions of service, so this is guided by the literature available [38,39]. It was estimated that on average 3 visits would be required following a fracture and 2 visits following other injuries in the base case analysis. Standardised prices or charges were applied to resources used. Whilst using charges instead of actual costs may not reflect the true opportunity cost of resources, applying standardised charges is more relevant for the healthcare system perspective and improves generalisability. Standardised prices used include Commonwealth Medicare Benefits Schedule (CMBS) [40] fees for General Practitioner consultations, an average of ambulance service costs obtained from the Private Health Insurance Administration Council (PHIAC) [41], Australian Ambulatory Classes [42] for Emergency Department visits, Australian Refined Diagnostic-Related Group (AR-DRG) cost weights [43] per hospital admission, and the Victorian Casemix Rehabilitation and Funding Tree (CRAFT) [44] for rehabilitation costs.

### Sensitivity analysis

A Monte Carlo Simulation was utilised in a probabilistic sensitivity analysis, and a cost-effectiveness acceptability curve constructed to indicate the probability of cost-effectiveness at any given value of willingness-to-pay [16]. 10,000 trials were run per simulation, with 56 uncertain variables covering the probabilities of each outcome, resource use and utility (Additional file 3: Table S3). The discount rate of costs and consequences was varied to 0 and 5% in the sensitivity analysis to allow broader interpretation of the results.

A threshold analysis was conducted to ascertain the falls rate reduction required to reach a cost-effectiveness threshold of GBP£20,000 to £30,000 per QALY [45] or to break even (cost-neutral). In further sensitivity analyses key inputs for program implementation were varied including the use of a fitness instructor, no opportunity cost of the venue or equipment and inclusion of local newspaper advertising. A gross outpatient cost incorporating Allied Health, General Practitioner consultations, specialists and pharmaceuticals was applied to investigate the effect of their exclusion on the ICER. The overall estimate was based on previously published cost estimates of falls [36,37].

The effect of missing falls surveillance data was explored in sensitivity analyses. In the most-likely scenario it was assumed that, in the absence of any intervention, participants with missing data would continue to fall at the baseline falls rate. In a worst-case scenario, it was assumed no change for exercise group participants with missing data, and no falls experienced by routine care group with missing data, and the reverse in a best-case scenario. The 17 participants who were randomised but did not commence the trial were included in this analysis.

## Results

### Effectiveness

Altogether there were 1448 falls recorded by the 1090 participants in the trial, 803 resulting in an injury. The rate of falls per year in the exercise group was 0.309 compared to 0.390 in the routine activity group (IRR:0.79, 95% CI 0.67 to 0.94) [15]. The injurious fall incidence rate ratio was 0.85 (95% CI 0.70 to 1.04) [15], just failing to reach statistical significance most likely as the RCT was powered to detect a difference in the falls rate rather than the injurious falls rate. However when a fall occurred there was compelling evidence showing no significant difference in the rate of injury in the routine activity compared to the exercise group (combined group: IRR 0.962, 95% CI: 0.76 to 1.21; women only group: IRR 0.989, 95% CI: 0.75 to 1.23). Subsequently a linear relationship was assumed between falls and injury, falls data pooled and equal transition probabilities applied to both groups after a fall (Additional file 4: Table S4). There was no significant difference in the falls rate reduction in the women only analysis compared to the overall group. However compared to women, men had a 44% lower rate of injury when they fell (IRR 0.56, 95% CI: 0.44 to 0.72).

Of all falls 55.5% resulted in injury. Fractures resulted from 2.5% of falls (3.7% for women). Altogether 36 fractures were recorded, 6 of which were hip fractures. 21.7% of injurious falls required medical care, and 3% of injuries required hospital admission. The most common injury sustained was a cut, scrape or bruise (73.2% of all injury), 91% of which did not require medical care.

### Costs

In the base case analysis with an AHA instructor the class cost £52.37 per participant, assuming 15 participants per instructor. In the sensitivity analysis this was varied to £36.09 with AHA instructor excluding venue hire and annual equivalent cost of equipment, £45.52 employing a fitness instructor or £29.24 excluding venue hire and annual equivalent cost of equipment. Non-discounted effects and costs per individual are presented in Table 2. As women are more likely to sustain fall-related injuries the cost of a fall and associated utility loss is higher for women. The estimated cost of each health state (excluding program cost) applied to the decision tree model is presented in Table 3.

**Table 2:** **Non-discounted individual costs and effects, “NoFalls” exercise program**

**Group**	**Effects**				**Costs (2010 GBP)**
	**QALY**	**Probability fall**	**Probability injurious fall**	**Probability fracture**	
**Exercise Program**	1.49530	0.371	0.206	0.009	£32.61 + program cost
Women only	1.49006		0.226	0.014	£46.08 + program cost
**Routine Activity**	1.49438	0.443	0.246	0.011	£38.94
Women only	1.48813		0.270	0.016	£55.03

**Table 3:** **Estimated cost of health states (excluding program cost)**

**Health state**	**Cost (GBP 2010)**
Hip fracture	£6611
Shoulder fracture - inpatient care	£8224
- ambulatory care	£46
Wrist fracture - inpatient care	£3,219
- ambulatory care	£46
Other fracture - inpatient care	£4762
- ambulatory care	£60
Cut / scrape / bruise - inpatient care	£1645
- ambulatory care	£39
Head injury - inpatient care	£2303
- ambulatory care	£75
Other injury - inpatient care	£4790
- ambulatory care	£48

### Cost-effectiveness

A summary of incremental cost-effectiveness ratios (ICERs) is shown in Table 4. The overall base case incremental cost per QALY is well above the accepted cost-effectiveness threshold. In the best-case scenario with a Fitness Instructor and minimised program implementation costs it is possible for the incremental cost per QALY to fall within the acceptable range. For women only the ICER is more favourable with all analyses falling within or below the cost-effectiveness threshold.

**Table 4:** **Summary ICER and sensitivity analysis, “NoFalls” exercise program**

**Cost scenario**	**GBP (2010)**	
	**Mixed gender**	**Women only**
***Effect:*** *Incremental QALYs 0.0009/0.0019 (women only)*	**Incremental cost per QALY (Value at 95% Confidence)**	
AHA - base case (incremental cost £45.87/£43.31 - women only)	£51483 (£99664)	£22986 (£48212)
AHA - no venue and minimal equipment cost (incremental cost £29.68/£27.13 - women only)	£33316 (£65218)	£14397 (£30373)
Fitness instructor – base case (incremental cost £ 39.06/£36.51 - women only)	£43845 (£84399)	£19375 (£41002)
Fitness instructor – no venue and minimal equipment cost (incremental cost £22.88/£20.32 - women only)	£25678 (£50649)	£10786 (£23645)
***Effect:*** *Incremental falls averted 0.0703 (mixed gender and women only)*	**Incremental cost per fall averted**	
AHA - base case	£652	£616
AHA - no venue and minimal equipment cost	£422	£386
Fitness instructor – base case	£556	£519
Fitness instructor – no venue and minimal equipment cost	£331	£289
***Effect:*** *Incremental injurious falls averted 0.039/0.043 (women only)*	**Incremental cost per injurious fall averted**	
AHA - base case	£1176	£1011
AHA - no venue and minimal equipment cost	£761	£633
Fitness instructor – base case	£1,002	£853
Fitness instructor – no venue and minimal equipment cost	£596	£475
***Effect:*** *Incremental fractures averted 0.0017/0.0026 (women only)*	**Incremental cost per fracture averted**	
AHA - base case	£26236	£16581
AHA - no venue and minimal equipment cost	£16978	£10385
Fitness instructor – base case	£22343	£13976
Fitness instructor – no venue and minimal equipment cost	£13294	£7780

### Sensitivity analysis

Incorporating advertising costs has an impact on the ICER, but does not significantly alter cost-effectiveness. Increasing costs associated with ambulatory care according to published cost estimates had minimal effect on the ICER. This indicates that excluding outpatient services such as Allied Health, specialists and pharmaceuticals has not substantially altered results (Additional file 5: Table S5).

**Figure 2:**
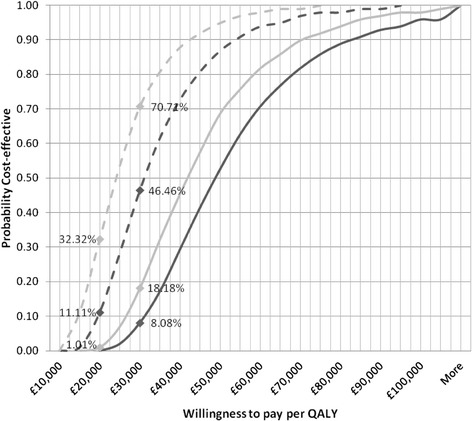
**CEAC - probability of cost-effectiveness at given value of willingness to pay, “NoFalls” Exercise Program (markers at GBP£20,000 and £30,000 per QALY).**  AHA: Base Case.  AHA: No Venue and minimal equipment cost.  Fitness Instructor: Base Case. Fitness Instructor: No venue and minimal equipment cost.

**Figure 3:**
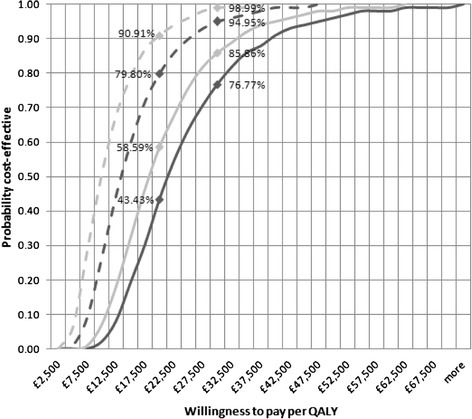
**CEAC - women only analysis, “NoFalls” Exercise Program (markers at GBP£20,000 and £30,000 per QALY).**  AHA: Base Case.  AHA: No Venue and minimal equipment cost.  Fitness Instructor: Base Case.  Fitness Instructor: No venue and minimal equipment cost.

The cost-effectiveness acceptability curves (CEAC) for the incremental cost per QALY are presented in Figures 2 and 3. The probability of reaching the accepted cost-effectiveness threshold of GBP£20,000 to £30,000 per QALY is extremely low for the overall base case utilising an AHA or Fitness Instructor, but much more encouraging for women only.

#### Threshold analysis

To fall within the cost-effectiveness threshold in the overall base case, the exercise program required a falls rate reduction of between 32% and 42%, assuming injury distribution remains constant. In the base case scenario employing a fitness instructor the falls rate reduction required is 28% to 37%. There is virtually no chance of the intervention breaking even or being cost-neutral, with a reduction in falls rate of over 80% required (61% for women only) in the best-case scenario.

#### Missing data sensitivity analysis

The falls rate reduction remains statistically significant under all scenarios explored in the missing data analysis (Additional file 6: Table S6). When the baseline falls rate is applied to all missing data there is little impact on the ICER. Apart from some of the best-case analyses, the overall ICER generally remains well above the cost-effectiveness threshold. Under most conditions the ICER remains within or below the cost-effectiveness threshold for women only analyses. As the injury data was pooled and applied equally to both groups missing data may weaken the results if fall-related injuries have been underestimated but would not cause bias.

## Discussion

Based on this evaluation there is little evidence to suggest that a group-based exercise program is more cost-effective than routine care to prevent falls among the older community-dwelling population. However there is evidence to support the program if offered to women only. This is driven by the higher probability of women sustaining a fracture in a fall resulting in higher costs and disutility, as well as the higher probability of women developing fear of falling and its associated disutility following a fall.

Direct comparison of this economic evaluation to other studies is difficult due to methodological differences in perspective, time frame and cost inclusions, as well as varied contexts within overseas health systems. Nevertheless a systematic review of economic evaluations of falls prevention interventions [6] identified three cost saving interventions in subgroups of high falls risk participants. These were a targeted multi-factorial intervention in the USA [46], the home-based Otago exercise program for people aged over 80 years in New Zealand [9] and a home safety program targeting those who had previously fallen and were discharged from hospital in Australia [47]. Another UK based study investigating the cost-effectiveness of cataract surgery for falls prevention in women reported an incremental cost per QALY well below the cost-effectiveness threshold when modelled over a lifetime [48].

An Australian based economic evaluation by Church et al. [12] utilising the effectiveness data from a systematic review by the Cochrane Collaboration [13] identified that group-based exercise was only cost-effective in a high risk population. The overall falls rate reduction of 22% (IRR: 0.78, 95% CI 0.71-0.87) utilised for group-based exercise is consistent with the falls rate reduction of 21% found in the “NoFalls” trial (IRR:0.79, 95% CI 0.67 to 0.94) [15]. Despite methodological differences the results of this evaluation support the findings of Church et al. in that group-based exercise programs in the general population would not be considered cost-effective. However, group-based exercise in the study by Church et al. and in variations of program implementation in this evaluation are approaching cost-effectiveness. Both evaluations indicate that in order to be cost-effective group-based exercise programs need to target sections of the older community-dwelling population.

Although the percentage of falls resulting in injury from this trial was consistent with previous reports [49,50] the proportion of fractures following a fall was lower than others reporting between 6% and 13% [35,49,50]. This may be due to differences in study populations, missing data or variations in definitions and methods used to record injurious falls [51], and could result in an underestimation of the cost of falls and therefore cost-effectiveness. The “NoFalls” trial was also powered to detect a difference in the rate of falls and had insufficient power to detect differences in less frequent injuries such as fractures. However it seems a reasonable assumption that a 15 week exercise program would not alter the fracture rate if a fall occurred. The large variation in the value of the ICER observed in the probabilistic sensitivity analysis was contributed to by the small numbers of more serious injuries such as fractures and infrequency of inpatient care.

There are some limitations which should be considered. There were differences between the study population and the general community-dwelling older population which may limit the generalisability of the results to people who are Australian born, aged 70–84 and of good to excellent health [14]. The lack of culturally diverse study populations is not unusual in falls prevention literature [2]. This economic evaluation was not planned for the “NoFalls” trial so data collection was not optimal. Sourcing utility values from the literature may have introduced some inaccuracy as these were derived from other study populations, although matched as best possible. Literature based utility estimates for fall-related injuries are also limited. Mortality, lifetime costs of falls such as nursing home placement or other longer term injury complications, and societal costs to families and friends providing support following falls have not been included. Conservative assumptions regarding utilities, time horizons and costs applied to this model potentially result in underestimation of true cost-effectiveness.

The cost-utility analysis is a strength of this evaluation as it allows broader comparison to other healthcare programs thereby facilitating decision making. In addition, fear of falling was incorporated as an important consequence of falling known to have a significant impact on the health-related quality of life of the older population. This study makes a contribution to the limited literature on trial based economic evaluations of group-based exercise programs for falls prevention. Whilst the limitations of the data available from the “NoFalls” trial have been acknowledged, this study has less potential for inaccuracies than those fully reliant on assumptions based on published literature and expert opinion.

Further research is required with larger sample sizes to enable more accurate observation of less frequent endpoints, over longer time frames to capture the full impact of fall-related injuries and fear of falling on utility. Measuring and reporting injurious falls using standardised methodology will also further enhance the accuracy and comparability of future research [51]. Standardising methodology and improving the accuracy of results will better inform budgetary decision-making.

## Conclusion

Falls and fall-related injury significantly impact on the health-related quality of life of older people and pose a considerable burden on the healthcare system. Although group-based exercise programs have proven effectiveness in reducing the falls rate in the older community-dwelling population, this economic evaluation provides little evidence to support its cost-effectiveness in a group of mixed gender. However the evidence does suggest that a group-based exercise program is cost-effective in an older female community-dwelling population. Group-based exercise programs aimed at falls prevention in the older community-dwelling population are more likely to provide value for money when targeted at women only due to their higher likelihood of fall-related injury and fear of falling. Group-based exercise programs are potentially a valuable component of broader inter-sectoral strategies for falls prevention and improved health in the community and warrant consideration as part of an Active Ageing policy.

## Additional files

Additional file 1: Table S1. Costs of program implementation, “NoFalls” Exercise Program. Additional file 2: Table S2. Costs of falls. Additional file 3: Table S3. Uncertain Variable Summary Information for probabilistic sensitivity analysis, “NoFalls” Exercise Program. Additional file 4: Table S4. Conditional probabilities applied over an 18-month period, “NoFalls” Exercise Program. Additional file 5: Table S5. Incremental cost per QALY (2010 GBP), “Nofalls” Exercise Program. Additional file 6: Table S6. Missing data analysis, “NoFalls” Exercise Program.
