# Supplementary material for: Economic evaluation of a group-based exercise program for falls prevention among the older community-dwelling population
Source: BMC Geriatr. 2015 Mar 26;15:33. doi: 10.1186/s12877-015-0028-x (PMC4404560; doi:10.1186/s12877-015-0028-x)
Supplement: Additional file 2: Table S2. — Costs of falls. [file 12877_2015_28_MOESM2_ESM.docx]

Additional file 2: Table S2: Costs of falls (currency conversion using PPP)
