# Supplementary material for: Economic evaluation of a group-based exercise program for falls prevention among the older community-dwelling population
Source: BMC Geriatr. 2015 Mar 26;15:33. doi: 10.1186/s12877-015-0028-x (PMC4404560; doi:10.1186/s12877-015-0028-x)

Additional file 3: Table S3: Uncertain Variable Summary Information for probabilistic sensitivity analysis, "NoFalls" Exercise Program.


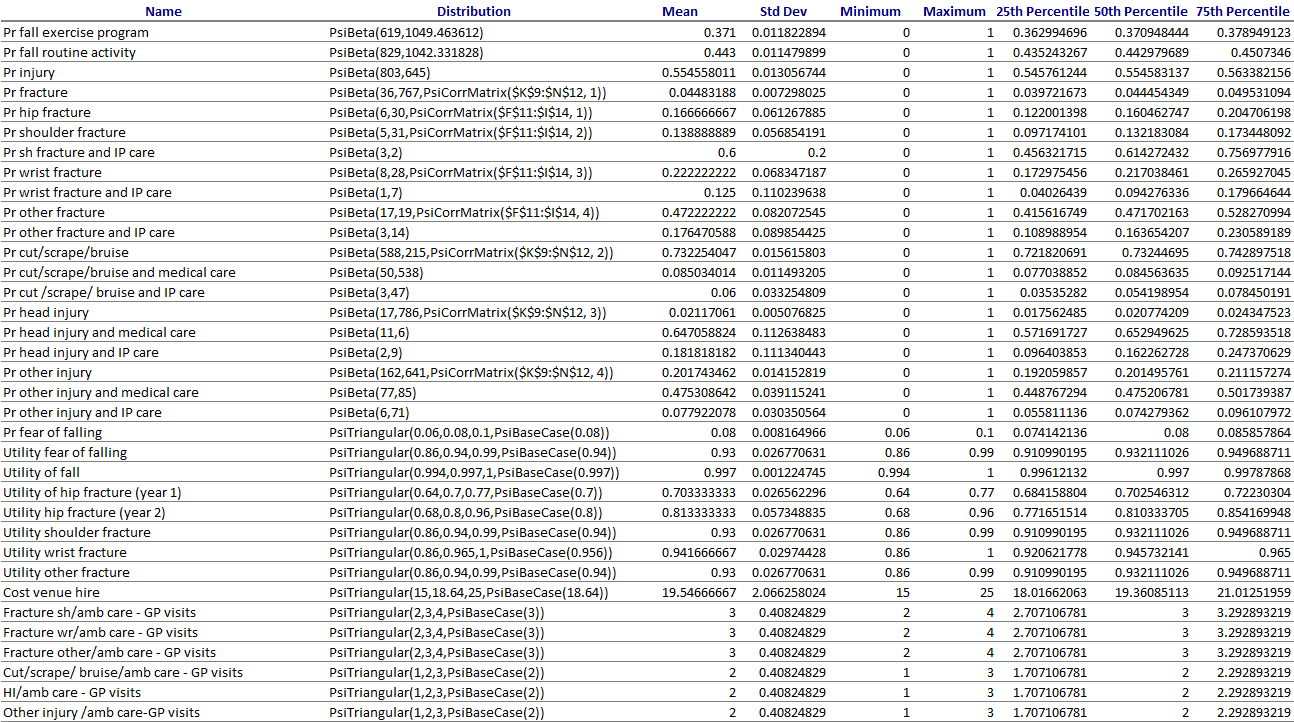


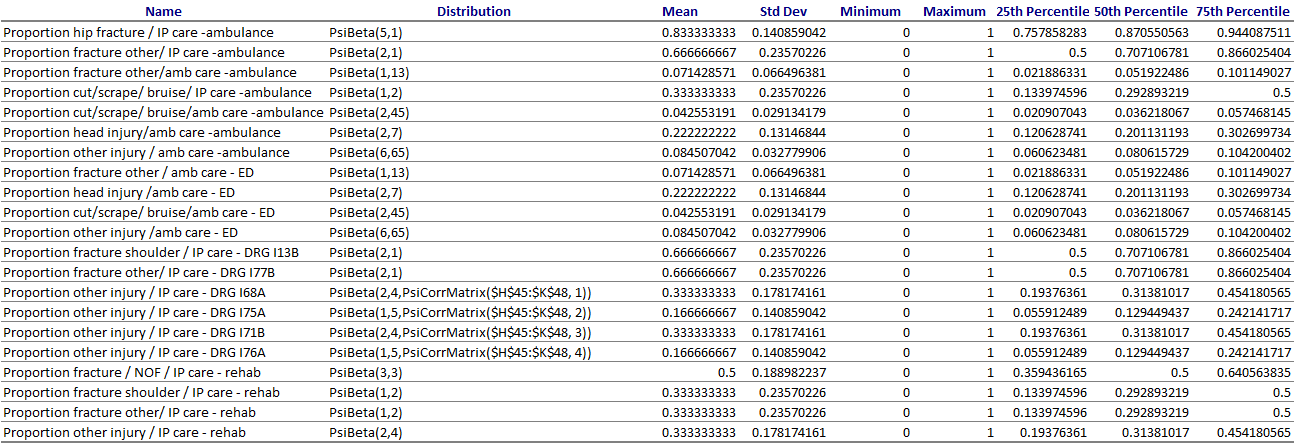

Supplement: Additional file 3: Table S3. — Uncertain Variable Summary Information for probabilistic sensitivity analysis, “NoFalls” Exercise Program. [file 12877_2015_28_MOESM3_ESM.docx]
