# Supplementary material for: Economic evaluation of a group-based exercise program for falls prevention among the older community-dwelling population
Source: BMC Geriatr. 2015 Mar 26;15:33. doi: 10.1186/s12877-015-0028-x (PMC4404560; doi:10.1186/s12877-015-0028-x)
Supplement: Additional file 4: Table S4. — Conditional probabilities applied over an 18-month period, “NoFalls” Exercise Program. [file 12877_2015_28_MOESM4_ESM.docx]

Additional file 4: Table S4: Conditional probabilities applied over an 18-month period, "NoFalls" Exercise Program

| **Branch** | **Value**  **(complete group)** | **Value (women only)** | **Source** |
| --- | --- | --- | --- |
| **Exercise Program** - Fall | 0.371 | 0.371 | "NoFalls" trial data  (unpublished) |
| - No Fall | 0.629 | 0.629 |  |
| **Routine Activity**  - Fall | 0.443 | 0.443 |  |
| - No Fall | 0.557 | 0.557 |  |
| **Exercise Program / Routine Activity** | | | |
| No Injury | 0.445 | 0.391 | Pooled "NoFalls" trial data  (unpublished) |
| Injury | 0.555 | 0.609 |  |
| Fracture | 0.045 | 0.061 |  |
| Neck of femur (NOF) | 0.167 | 0.176 |  |
| Shoulder | 0.139 | 0.118 |  |
| IP care | 0.6 | 0.75 |  |
| Ambulatory care | 0.4 | 0.25 |  |
| Wrist | 0.222 | 0.206 |  |
| IP care | 0.125 | 0.14 |  |
| Ambulatory care | 0.875 | 0.86 |  |
| Other | 0.472 | 0.5 |  |
| IP care | 0.176 | 0.18 |  |
| Ambulatory care | 0.824 | 0.82 |  |
| Cut / scrape / bruise | 0.732 | 0.701 |  |
| Medical care | 0.09 | - |  |
| IP care | 0.94 | - |  |
| Ambulatory care | 0.06 | 0.079 |  |
| No medical care | 0.91 | 0.921 |  |
| HI | 0.021 | 0.023 |  |
| Medical care | 0.647 | 0.615 |  |
| IP care | 0.182 | 0.125 |  |
| Ambulatory care | 0.818 | 0.875 |  |
| No medical care | 0.353 | 0.385 |  |
| Other | 0.202 | 0.215 |  |
| Medical care | 0.475 | 0.492 |  |
| IP | 0.078 | 0.0678 |  |
| Ambulatory care | 0.922 | 0.9322 |  |
| No medical care | 0.525 | 0.508 |  |
| Fear of falling (FOF) after a fall | 0.08 | 0.23 | Freidman^[26]^ /  Murphy^[27]^ |
