# Supplementary material for: Economic evaluation of a group-based exercise program for falls prevention among the older community-dwelling population
Source: BMC Geriatr. 2015 Mar 26;15:33. doi: 10.1186/s12877-015-0028-x (PMC4404560; doi:10.1186/s12877-015-0028-x)
Supplement: Additional file 6: Table S6. — Missing data analysis, “NoFalls” Exercise Program. [file 12877_2015_28_MOESM6_ESM.docx]

| **Scenario for sensitivity analysis** | **IRR**  **(95% confidence interval)*** | **Incremental cost per QALY (GBP 2010)** | | |
| --- | --- | --- | --- | --- |
|  |  | **Cost scenario** | **Mixed gender** | **Women only** |
| ***Most likely scenario:*** participants with missing data maintained their baseline falls rate | | | | |
| Participants in both groups assigned pre-intervention falls rate for each month missing (pre-intervention falls rate of 0.08 per month recorded at baseline) | 0.795  (0.68 - 0.93) | AHA - base case | £54,023 | £24,187 |
|  |  | AHA - no venue and minimal equipment cost | £35,066 | £15,224 |
|  |  | Fitness instructor – base case | £46,053 | £20,419 |
|  |  | Fitness instructor – no venue and minimal equipment cost | £27,096 | £11,456 |
| ***Worst case scenario:*** no change for exercise group participants with missing data, and no falls experienced by routine care group with missing data | | | | |
| Participants in the routine care group assigned no falls for each month missing and participants in exercise group assigned pre-intervention falls rate for each month missing. | 0.856  (0.73 - 0.92) | AHA - base case | £84,503 | £38,597 |
|  |  | AHA - no venue and minimal equipment cost | £56,068 | £25,154 |
|  |  | Fitness instructor – base case | £72,548 | £32,945 |
|  |  | Fitness instructor – no venue and minimal equipment cost | £44,113 | £19,501 |
| ***Best case scenario:*** no falls experienced by exercise group participants with missing data, and no change for routine care group with missing data | | | | |
| Participants in the exercise group assigned no falls and participants in routine care group assigned pre-intervention falls rate for each month missing. | 0.696  (0.6 - 0.81) | AHA - base case | £33,122 | £14,305 |
|  |  | AHA - no venue and minimal equipment cost | £20,665 | £8,415 |
|  |  | Fitness instructor – base case | £27,885 | £11,829 |
|  |  | Fitness instructor – no venue and minimal equipment cost | £15,428 | £5,939 |

Additional file 6: Table S6: Missing data analysis, "NoFalls" Exercise Program

*Reported base IRR 0.79 (0.67 - 0.94)
